# Needle-free technique for guidewire manipulation during endoscopic ultrasound-guided pancreatic duct drainage

**DOI:** 10.1055/a-2261-7735

**Published:** 2024-02-22

**Authors:** Takeshi Ogura, Masahiro Yamamura, Mitsuki Tomita, Jun Sakamoto, Hiroki Nishikawa

**Affiliations:** 1 Endoscopy Center, Osaka Medical and Pharmaceutical University Hospital, Osaka, Japan; 2 2nd Department of Internal Medicine, Osaka Medical and Pharmaceutical University, Osaka, Japan

Endoscopic ultrasound-guided pancreatic duct drainage (EUS-PD) is considered if the pancreatic duct approach under endoscopic retrograde cholangiopancreatography guidance is unsuccessful due to failure of pancreatic duct cannulation or an inaccessible papilla 1 2 3 . During EUS-PD, guidewire manipulation may be one of the limiting steps, especially in nonexpert hands 4 . Guidewire manipulation may fail due to the guidewire shearing against the needle. During EUS-guided hepaticogastrostomy, to prevent guidewire shearing, the liver impaction technique can be attempted 5 ; however, during EUS-PD, the short length of pancreatic parenchyma on the puncture route may render this technique challenging. To overcome this, technical tips for a needle-free technique during EUS-PD are described.

A 71-year-old man was admitted to our hospital with stricture of the pancreatojejunostomy. As the enteroscopic approach had failed, EUS-PD was attempted. First, the main pancreatic duct was punctured using a 19-gauge needle, and then a 0.025-inch guidewire with an angle tip was inserted; however, the guidewire was advanced into the pancreatic tail instead of the head ( Fig. 1 ). Attempts were made to change direction by pulling the guidewire; however, this was unsuccessful because of shearing against the needle. In addition, the short length of pancreatic parenchyma on the puncture route meant that the impaction technique could not be performed. Therefore, the needle was first completely retracted into the needle sheath ( Fig. 2 ). By doing so, the tip of the needle was protected by the sheath and guidewire shearing could not occur. After this procedure, it was possible to manipulate the guidewire easily and smoothly. The guidewire was pulled gently ( Fig. 3 **a** ) and successfully advanced toward the stricture site ( Fig. 3 **b** ). After tract dilation, a 7-Fr plastic stent was successfully deployed without any adverse events ( Video 1 ).

**Fig. 1:**
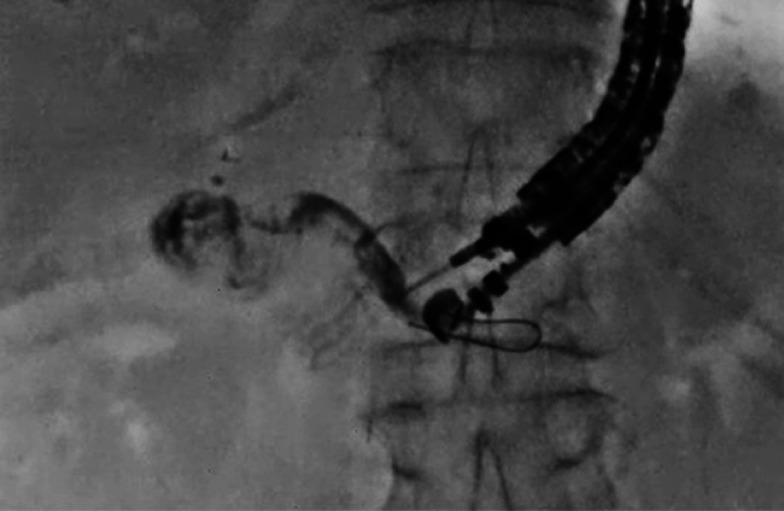
The guidewire was advanced into the pancreatic tail.

**Fig. 2:**
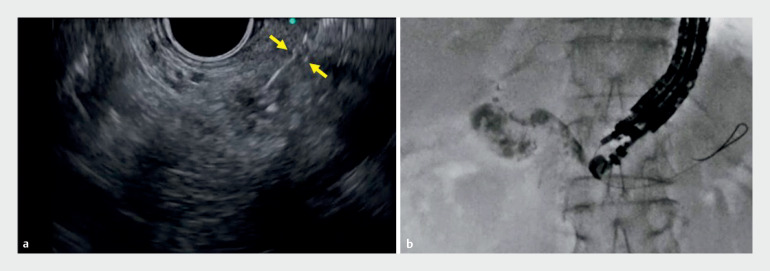
To prevent guidewire shearing, the needle was completely retracted into the needle sheath (arrow). **a** Endoscopic ultrasound guidance. **b** Fluoroscopic guidance.

**Fig. 3:**
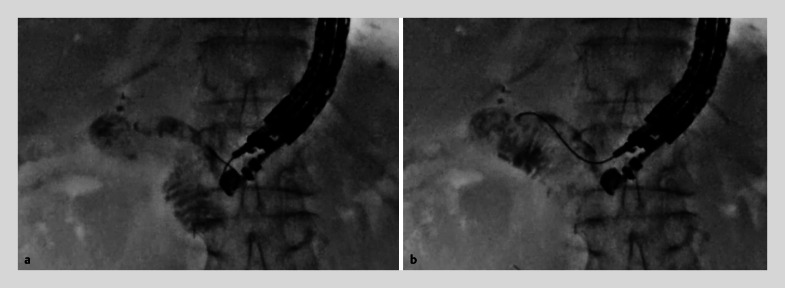
Fluoroscopic images. **a** Guidewire manipulation was performed smoothly. **b** Guidewire deployment into the head of the pancreas was successfully performed.

Needle-free technique for guidewire manipulation during endoscopic ultrasound-guided pancreatic duct drainage.Video 1

In conclusion, the present technique might be useful for guidewire manipulation during EUS-PD.

Endoscopy_UCTN_Code_TTT_1AS_2AD

## Notes

Endoscopy E-Videos https://eref.thieme.de/e-videos
*E-Videos* is an open access online section of the journal *Endoscopy* , reporting on interesting cases and new techniques in gastroenterological endoscopy. All papers include a high-quality video and are published with a Creative Commons CC-BY license. Endoscopy E-Videos qualify for HINARI discounts and waivers and eligibility is automatically checked during the submission process. We grant 100% waivers to articles whose corresponding authors are based in Group A countries and 50% waivers to those who are based in Group B countries as classified by Research4Life (see: https://www.research4life.org/access/eligibility/ ). This section has its own submission website at https://mc.manuscriptcentral.com/e-videos .
